# A Caregiver-Child Intervention for Mitigating Toxic Stress (“The Resiliency Clinic”): A Pilot Study

**DOI:** 10.1007/s10995-022-03485-4

**Published:** 2022-08-10

**Authors:** Joan Jeung, Danielle Hessler Jones, Laura Frame, Rachel Gilgoff, Dayna Long, Neeta Thakur, Kadiatou Koita, Monica Bucci, Nadine Burke Harris

**Affiliations:** 1 University of California San Francisco (UCSF) School of Medicine, 675 18th St, 94143 San Francisco, CA United States; 2 UCSF Benioff Children’s Hospital Oakland, Oakland, United States; 3 Aurrera Health Group, Sacramento, United States; 4 California Office of the Surgeon General, Sacramento, United States; 5 Center for Youth Wellness/Safe and Sound, San Francisco, United States

**Keywords:** Adverse childhood experiences, Parent education, Pediatric primary care, Toxic stress, Group visit

## Abstract

**Introduction:**

Primary care-based interventions that promote nurturing caregiving relationships and early relational health may help mitigate toxic stress and promote resilience in children. This pilot study aims to: (1) describe a novel group-based, psychoeducational primary care intervention for children experiencing adverse childhood experiences (ACEs) (“The Resiliency Clinic”), (2) assess program feasibility and acceptability, and (3) explore effects on child/caregiver behavioral health.

**Methods:**

Intervention design centered on promoting supportive caregiving, caregiver/child self-regulation and co-regulation and teaching evidence-based stress management tools. Program feasibility and acceptability were assessed through attendance data and caregiver focus groups. Behavioral health measures were obtained at baseline and 8-month follow-up.

**Results:**

Of 101 eligible families, 38 (37.6%) enrolled and attended a median of 3.00 (mean = 2.95, sd = 1.75) out of 6 sessions. Caregivers reported high satisfaction and benefits including stress management tools and connection with staff and other parents. There were modest, statistically non-significant improvements in caregiver stress (*d* = 0.23) and child executive functioning (*d* = 0.27).

**Discussion:**

In conclusion, a group intervention teaching supportive caregiving and stress mitigation is feasible and acceptable for many families in an urban federally qualified health center (FQHC) with a signal for modest improvements in behavioral health. Future program iterations will seek to address participation barriers and expand the intervention’s capacity to promote early relational health.

## Significance

*What is already known about this subject?* Toxic stress resulting from childhood adversity can increase risk for behavioral, developmental, and health disorders, but nurturing relationships can “buffer” stress and promote resilience in children. Pediatric medical homes are well-positioned to mitigate toxic stress, but little research addresses primary care interventions for adverse childhood experiences (ACEs).

*What this study adds*: This pilot study describes and evaluates a new primary care-based, group intervention for children ages 0–11 years exposed to ACEs. Our study supports this intervention’s feasibility and acceptability in primary care and provides preliminary estimates of its efficacy to inform future research.

## Introduction

Toxic stress occurs when prolonged exposure to adversity over-activates the body’s stress response in the absence of protective factors (Shonkoff et al., 2012), increasing risk for learning disorders, mental illness, substance abuse, and cardiometabolic disease (Felitti et al., 1998; Lupien et al., 2009; Miller et al., 2011). Nurturing relationships with adult caregivers can “buffer” children from adversity, making early relational health a key priority in pediatrics (Garner & Yogman, 2021). The pediatric medical home is well-positioned to address toxic stress given its universal and non-stigmatized contact with children (Garner et al., 2012). However, a systematic review of interventions addressing pediatric adverse childhood experiences (ACEs) found little research involving primary care settings (Marie-Mitchell & Kostolansky, 2019).

This is a pilot study of the Resiliency Clinic, a primary care intervention for caregivers/children ages 0–11 years exposed to ACEs. This program aims to promote child resilience by: (1) teaching evidence-based stress mitigation strategies and (2) strengthening supportive caregiving practices. This pilot study seeks to: (1) describe this novel intervention, (2) assess its operational feasibility and acceptability in an urban federally qualified health center (FQHC), and (3) explore preliminary estimates of program effect on child and caregiver behavioral health.

## Methods

### Intervention Design and Implementation

#### Theory of Change & Program Design

**Fig. 1:**
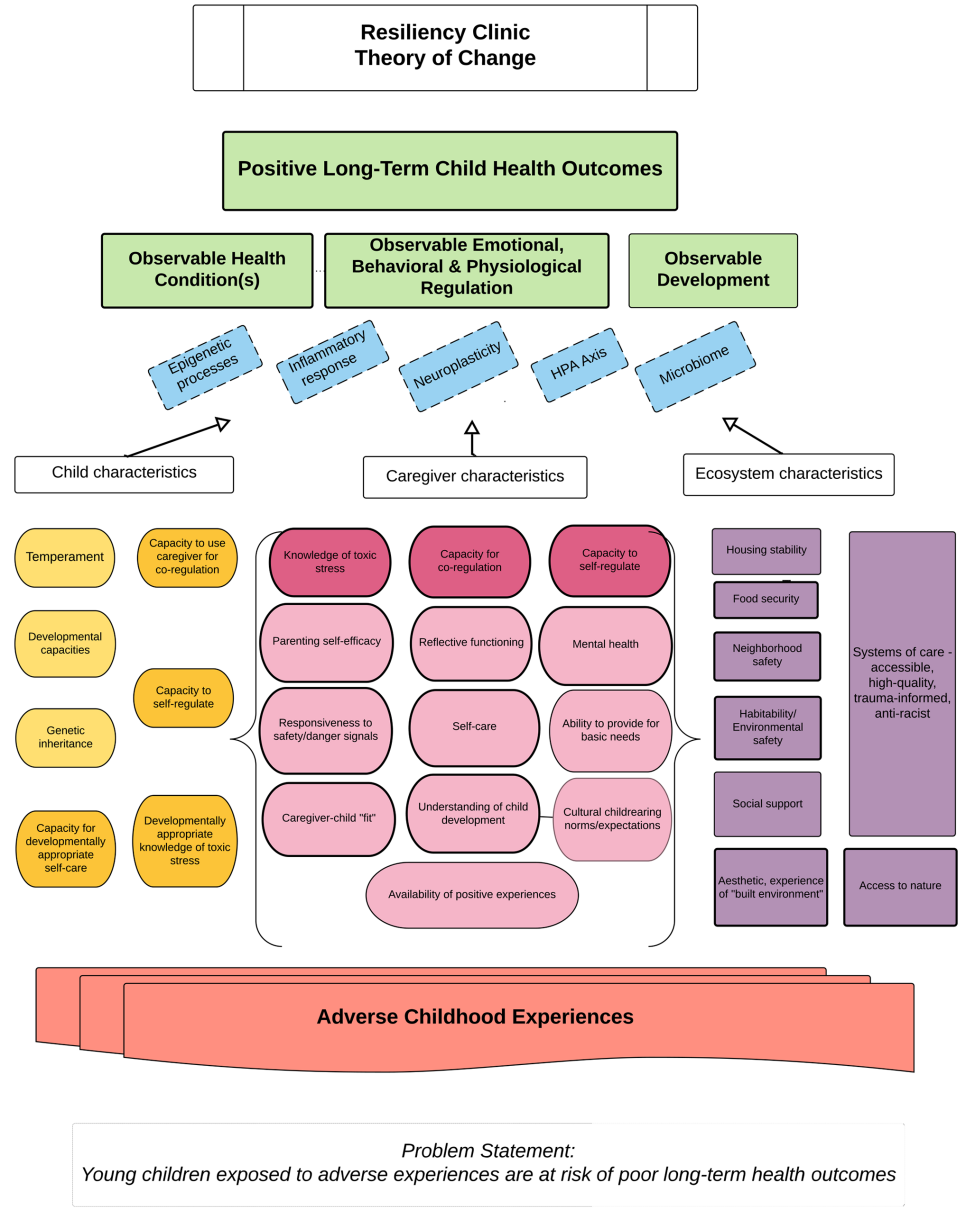
Resiliency Clinic Theory of Change

Using a Theory of Change (TOC) framework, a multi-disciplinary team engaged in a series of meetings to articulate long-term goals and backward-map these goals to pre-conditions in a causal framework (Taplin & Rasic, 2012). This team of researchers, psychotherapists, pediatricians, and trauma survivors reviewed relevant research, identified age-appropriate stress-mitigation tools, and mapped activities required to attain each goal. The final TOC framework is presented in Fig. 1.

Starting from the bottom, Fig. 1 outlines how ACEs affect child health outcomes through a set of preconditions (grouped into child, caregiver, and ecosystem characteristics) via mediating physiological processes. The diagram’s preconditions represent possible intervention targets. Of these, the team selected the capacity for self-regulation and caregiver-child co-regulation; knowledge of toxic stress; and capacity for self-care to address in the program.

A key target was self-regulation, which can be strengthened by mindfulness or the capacity to attend to the present moment without judgment (Kabat-Zinn, 2005). An important caregiving task, which requires self-regulation, is helping a child modulate stress-related arousal through responses that are soothing, containing, and organizing (Schore & Schore, 2014). This process of “co-regulation”(Tronick & Gianino, 1986) helps young children develop self-regulation capacity (Gunnar & Donzella, 2002). Together, building self-regulation and co-regulation skills formed the crux of the Resiliency Clinic curriculum.

#### Program Structure as Implemented

The Resiliency Clinic used a group visit model in which 4–8 child-caregiver dyads met in six 2-hour, monthly group sessions. Cohorts stayed together for the 6-month period and were grouped by age: 0–2 years, 3 to 5 years, and 6 to 11 years.

Co-facilitated by a psychotherapist and medical provider, each visit featured a discussion of the session theme: (1) Stress, Health and Well-Being, (2) Linking Feelings, Regulation and Movement, (3) Relationships and Regulation, (4) Sensory Input, Regulation and Sleep, (5) Nutrition, Stress and Health, and (6) Nature and Well-Being. Afterwards, each dyad took turns visiting the medical provider while the others completed art projects introducing self-regulation tools like mindful jars, body sketches locating where stress/emotions were felt, feeling sticks, worry trolls, scented lotions, and succulent planting. The medical visit focused on stress-related health issues like headaches, abdominal pain, asthma, and behavior concerns. Afterwards children had a snack/story time while caregivers met with the therapist for discussion. Finally, caregivers and children returned for a closing activity. Families left with a workbook designed to reinforce lessons and share tools with friends and relatives.

### Research Methods

#### Pilot Study Population and Design

Data for this study was collected as part of the PEdiatric ACEs and ResiLiency (PEARLS) Study (Koita et al., 2018; Thakur et al., 2020), a 12-month study (NCT04182906) designed to validate a prospective pediatric screening tool for ACEs and related life events (the PEARLS) (Thakur et al., 2020) and pilot interventions for toxic stress. The study site was an FQHC serving a diverse urban population. Recruitment details for the larger study have been reported previously (Thakur et al., 2020). The present study focuses on the n = 38 families who were randomized to and participated in the resiliency intervention.

Child participants were eligible if they were 3 months to 11 years old; received primary care at the study site; and had a PEARLS score of at least one, signifying one or more ACEs/related life events. Caregivers were eligible if they were at least 18, the primary caregiver, and English or Spanish speaking. Foster children were excluded. Siblings were excluded from the study but were invited to participate in program activities. All participants provided written informed consent and the study was approved by the site’s Institutional Review Board. Participants received $300 for undergoing data collection procedures but were not incentivized to attend program sessions. Research staff randomized participants via an automated randomization table and administered the PEARLS tool, followed by additional psychological and health questionnaires at baseline and follow-up 8 months later.

Attendance records and caregiver focus groups were used to evaluate program feasibility and acceptability. All caregivers who attended at least one session were invited to participate in focus groups that queried their satisfaction with the program, lessons learned, benefits for themselves and their children, barriers to participation, and suggestions for change. Focus groups were conducted via a HIPAA-compliant virtual platform and followed a semi-structured protocol. With participants’ verbal permission, focus groups were recorded; recordings were uploaded to Microsoft Word (Office 365 Enterprise), which was used for automatic transcription. Detailed notes were also taken. Transcripts were reviewed, cleaned, and de-identified for analysis.

#### Measures

At baseline, participants reported on demographic information and child ACEs and Related Life Events using the PEARLS tool. The PEARLS is an adversity screening tool (Koita et al., 2018; Thakur et al., 2020) including the original ACE categories (child maltreatment, caregiver substance abuse or mental illness, inter-partner violence, divorce, and incarceration)(Felitti et al., 1998) as well as seven additional Related Life Events (e.g., food insecurity, housing instability, discrimination) (Thakur et al., 2020) thought to increase the risk of toxic stress (Koita et al., 2018; Shonkoff et al., 2012). At baseline and 8-month follow-up, caregivers reported their stress using the Perceived Stress Scale (PSS)(Cohen et al., 1983) with higher scores indicating greater stress (range 0–40; ≥ 14 moderate and ≥ 27 high stress). Caregivers also reported on their children’s behavioral health at baseline and follow-up using the Behavior Rating Inventory of Executive Function (BRIEF 2/P) tool(Sherman & Brooks, 2010) (higher Global Executive Composite scale t-scores indicate greater risk).

#### Data Analysis Plan

Qualitative analysis consisted of an iterative process of developing and modifying codes based on thematic data review and re-review of all narrative data (Maguire & Delahunt, 2017), and was conducted by two study investigators who independently reviewed the focus groups transcripts and developed codes based on the study aims, participant responses, and findings emerging from the data. Codes were tracked in the source documents using the comments feature in Microsoft Word. The qualitative data analysts then grouped their codes into overarching themes and discussed their findings, reconciling any differences through discussion and consensus. Findings are summarized and illustrated with quotes and examples. To ensure reliability and validity, at least two investigators reviewed coding schemes to ensure a shared understanding of their meaning (Miles et al., 2019; Padgett, 2016). Negative case analysis and checking exceptional data took place through a skeptical approach to emerging themes.

Quantitatively, descriptive statistics, chi-square tests, and one-way ANOVAs were computed for baseline measures, initially testing for differences between enrolled vs. non-enrolled participants and those with and without follow-up data. Dependent t-test analyses examined the impact of the intervention on outcomes via pre- vs. post- intervention change. Missing data were treated as missing (not imputed). Statistical analyses were performed using SPSS version 19.0 (IBM Corp., Chicago, IL). Linear regressions further explored constructs associated with improvement in the outcomes including: (1) demographic factors (child age, gender, race/ethnicity, income), number of PEARLS adversities, and number of intervention sessions attended (dose).

## Results

### Pilot Study Population Characteristics

Family demographic characteristics are displayed in Table 1. The population was predominantly non-Hispanic Black and low-income, with a mean age of 6.9 years for children and 38 years for caregivers. By study design, all caregivers reported at least one child adversity with a median report of 4 (interquartile range 1–5) adversities. Nearly two thirds (63.2%) of families completed follow-up measures. Neither enrollment nor attrition at follow-up were associated with family demographic characteristics or number of adversities.

**Table 1:** Baseline Characteristics of Pilot Resiliency Clinic Study Population

	N % Total N = 38
Child age, mean (SD)	6.85 (3.14)
Child gender	
Male	58.9% (22)
Female	42.1% (16)
Child race / ethnicity	
non-Hispanic Black	39.5% (15)
Hispanic	23.7% (9)
non-Hispanic White	7.9% (3)
Other	28.9% (11)
Caregiver age, mean (SD)	38.16 (10.19)
Caregiver gender	
Male	10.5% (4)
Female	89.5% (34)
Caregiver race / ethnicity	
non-Hispanic Black	52.6% (20)
Hispanic	23.7% (9)
non-Hispanic White	10.5% (4)
Other	13.2% (5)
Caregiver education	
Some high school or less	13.2% (5)
High school	18.4% (7)
Some college	39.5% (15)
College	28.9% (11)
Family income	
25,000 or less	59.5% (22)
More than 25,000	40.5% (15)
PEARLS pediatric screening score	
Original ACEs (median; IQR)	3; 1 - 4
Related life events (median; IQR)	1; 0 - 2
Total PEARLS score (median; IQR)	4; 2 - 5

### Feasibility & Acceptability

#### Attendance

Of the 101 eligible families who were offered the Resiliency Clinic, 38 (37.6%) enrolled and attended one or more sessions. Of these, 34% attended one, 36% attended between 2 and 4, and 29% attended 5–6 of the 6 sessions offered. The median number of sessions attended was 3.00 (mean = 2.95, sd = 1.75).

#### Qualitative data

A total of 8 caregivers who attended the Resiliency Clinic (range 1–6 visits) engaged in two focus groups. Nearly all recommended the Resiliency Clinic because of the sense of community it provided, the safe environment it created, and self-regulation tools. Some parents realized the importance of their own self-regulation in managing child behavior. Parents also reported that their school-age children learned new ways to talk about and manage their stressful experiences and emotions, strengthening parent/child communication. Participants also appreciated take-home materials and guided meditations.

The importance of relationships emerged as a key theme. Several caregivers underscored the importance of the relationships built with the therapist and medical provider. Others described how program activities strengthened their relationships with their children. This included a clearer understanding of their children’s experience of stress and how to support them with their feelings and experiences.

I feel that I got to know my son in a different way than just a mom … and kind of learn a little bit more about how he expresses his feelings … so I think it brought us a little closer together.

You think you know your children but you really don’t know them …. There have been incidents where they lock down the school. … We ended up taking the resilient class, (and) I found there are still nightmares… (my daughter) got caught up in a drive-by shooting …It was eye opening for me to learn her better and then we could work on things together with the skills they taught us.

Obstacles to attendance included competing priorities and limited caregiver time and resources, leading to inconsistent scheduling and canceled sessions. As one participant explained, “I had my hands full and wasn’t able to participate as much as I would have liked.” However, when they could meet, parents valued the chance to connect with others, as one parent explained: “We all just need someone to talk to and our kids need someone to talk to. We need a sense of community.”

### Pilot Resiliency Outcomes

Pre-post changes in caregiver perceived stress and measures of reported child health are summarized in Table 2.

**Table 2:** Pilot Resiliency Pre-post Child and Caregiver Outcomes

	Baseline	Follow-up	Change	p-value	Effect size
Perceived Stress Scale (Caregiver Perceived Stress)	17.70 (7.53)	15.46 (8.82)	-2.25 (9.47)	0.26	0.27
BRIEF Global Executive Composite Scale t-score (continuous)	56.79 (12.34)	53.74 (13.86)	-3.05 (8.18)	0.12	0.23

For the PSS, higher scores indicate higher stress (range 0-40). For the BRIEF, higher scores indicate greater risk.

The small sample size should be considered when interpreting results. While changes did not reach statistical significance, positive improvements were reported by caregivers that translated to statistical small effect sizes in caregiver stress (*d* = 0.23) and child executive functioning (*d* = 0.27). Family demographic factors, number of reported adversities, and number of attended sessions were not statistically significantly related to the degree of reported change in caregiver stress and child executive functioning. However, a positive association between number of sessions attended and decreases in caregiver perceived stress approached significance (unstandardized beta = 1.98, standardized beta = 0.34, *p* = .10).

## Discussion

The Resiliency Clinic is a primary care-based, caregiver-child group intervention designed to promote resilience in children exposed to ACEs. This pilot study aimed to describe this novel intervention and evaluate its feasibility and acceptability and explore its impact as implemented in an urban FQHC. Due to small sample sizes and lack of a comparison group, it is difficult to draw firm conclusions. However, this pilot still provides valuable lessons regarding the potential benefits and drawbacks of the caregiver/child psychoeducational group model in primary care, findings that may help others seeking to strengthen primary care capacity to support early relational health.

One lesson concerns group attendance for psychoeducational purposes. Only 37% of eligible dyads participated, attending a median of 3 out of 6 sessions, high enough to make it feasible in clinical settings, but not reaching many who were eligible. Because attendance was not incentivized, this pilot reflects “real-world” conditions showing the likely uptake of a voluntary, preventative group intervention in primary care. Logistical scheduling difficulties and heavy caregiver responsibilities were commonly cited as barriers. Like other group interventions (Frame et al., 2006; Jones et al., 2018), this pilot demonstrated that the group visit format was feasible and often valued by parents but faced logistical barriers hindering implementation.

This pilot also provides a preliminary estimate of program effect on child executive function and parent perceived stress (*d =* 0.27 and 0.25 respectively), a positive though modest impact, with a trend towards decreased parent perceived stress with increased attendance. Given the program’s small size and light touch, these initial estimates, along with parents’ positive feedback, suggest that this intervention provides a promising base for further innovation, and may help inform future research on programs with similar aims.

Those who did attend reported high acceptability, at least among the segment of caregivers in our focus groups. Focus group participants almost universally indicated that they would recommend the program to others, citing benefits like connecting with other parents, program staff, and their own children as well as gaining tools for stress management and parenting challenges. Our focus group participants viewed the group setting as a strength promoting interpersonal connections needed for resilience. The group model was chosen as an efficient way of delivering psychoeducation while offering support from peers and clinicians. Group medical visits have demonstrated positive outcomes for patients with diabetes and asthma (Housden et al., 2013; Wall-Haas et al., 2012). Prior research also suggests that multi-family, caregiver-child groups can effectively provide mental health care, partially through peer and professional support (Frame et al., 2006). This study’s qualitative findings, while based on a small group of participants, support the child-caregiver group visit model as a potentially effective way to deliver psychoeducation about stress and supportive caregiving.

Several study limitations impact its findings. First, the limited sample size limits its generalizability and statistical power. Second, the larger study in which this pilot was embedded lacked a usual care comparison group and contained a limited set of measures capturing more immediate program impacts. Third, lower attendance may have diminished impact. Areas for future research include ways to increase attendance, exploring whether other formats (home visits, virtual groups, community-based groups) are more accessible. Quality improvement research is also needed to improve the curriculum’s impact on intervention targets suggested by the Theory of Change (caregiver-child self-regulation and co-regulation skills). Future program iterations plan to leverage current strengths while adjusting activities and logistics based on this study.

In conclusion, a group intervention to teach stress-mitigation strategies and strengthen supportive caregiving is feasible and acceptable for a significant portion of families in an urban FQHC and demonstrates potential for improving caregiver and child behavioral health outcomes. Despite logistical challenges, group visits constitute a feasible and acceptable approach for a subset of families who can attend regular meetings and want to connect with peers. Future program iterations will seek to address participation barriers and expand the intervention’s capacity to promote early relational health.

## Data Availability

Quantitative and qualitative data reside with the study authors and can be made available upon request. No custom codes were developed for this study.
